# The Prevalence of Pretreatment Drug Resistance and Transmission Networks Among Newly Diagnosed HIV-1-Infected Individuals in Nanning, Guangxi, China

**DOI:** 10.3390/pathogens14040336

**Published:** 2025-03-31

**Authors:** Qiuqian Su, Yanjun Li, Ting Huang, Liangjia Wei, Jinfeng He, Yumei Huang, Guidan Mo, Jiao Qin, Chunxing Tao, Xinju Huang, Li Ye, Hao Liang, Bingyu Liang, Jinping Huang

**Affiliations:** 1 Guangxi Key Laboratory of AIDS Prevention and Treatment, School of Public Health, Guangxi Medical University, Nanning 530021, China; 2023989@sr.gxmu.edu.cn (Q.S.); 202320978@sr.gxmu.edu.cn (T.H.); 202210187@sr.gxmu.edu.cn (L.W.); hejinfeng@sr.gxmu.edu.cn (J.H.); 202420967@sr.gxmu.edu.cn (G.M.); qinjiao@sr.gxmu.edu.cn (J.Q.); 202320879@sr.gxmu.edu.cn (C.T.); 202220992@sr.gxmu.edu.cn (X.H.); yeli@gxmu.edu.cn (L.Y.); lianghao@gxmu.edu.cn (H.L.); 2 School of Public Health, Guangxi Medical University, Nanning 530021, China; 3 The Fourth People’s Hospital of Nanning, Nanning 530023, China; qq389585345@outlook.com (Y.L.); qsy2605989934@outlook.com (Y.H.); 4 Biosafety III Laboratory, Life Science Institute, Guangxi Medical University, Nanning 530021, China; 5 Department of Clinical Research, London School of Hygiene & Tropical Medicine, London WC1E 7HT, UK

**Keywords:** HIV/AIDS, pretreatment HIV-drug resistance, drug mutation, transmission networks, molecular epidemiology

## Abstract

The scale-up of antiretroviral therapy (ART) has markedly increased pretreatment drug resistance (PDR) among newly diagnosed HIV-infected individuals. This study aims to assess the prevalence and characteristics of PDR, infer the genetic transmission network, and evaluate the effect of PDR on ART in Nanning City, Guangxi. Methods: This study was conducted in the Fourth People’s Hospital of Nanning from 2019 to 2023. PDR was estimated using the Stanford algorithm. Genetic transmission networks were inferred by HIV-TRACE and visualized with Cytoscape. Logistic regression identified PDR-related factors. The Cox proportional hazards model assessed the impact of drug resistance on virological and immunological failure. Among 234 participants, the prevalence of PDR was 8.97%. CRF07_BC (35.9%), CRF-01AE (27.35%), and CRF08_BC (23.9%) were the top three *HIV-1* strains. Resistance to non-nucleoside reverse-transcriptase inhibitors, protease inhibitors, nucleoside reverse-transcriptase inhibitors, and integrase strand-transfer inhibitors was 4.27%, 2.56%, 1.28%, and 0.43%, respectively. Overall, 21.37% of the participants exhibited drug resistance mutations (DRMs). Homosexuals were less likely to have PDR compared to heterosexuals ([aOR] 0.09, 95% CI 0.01–0.86). In the genetic network, *V179D/E* was also the most frequent DRM. Additionally, the incidence of virological failure (19.23%) and immune failure (20.09%) after one year of treatment did not show significant differences in different drug resistance groups. Conclusions: The prevalence of PDR in Nanning City is moderate, driven largely by the *V179D* and *K103N* mutations. The cross-transmission networks emphasize the imperative of PDR testing and targeted interventions.

## 1. Introduction

By the end of December 2022, around 29.8 million people (76%) living with HIV/AIDS were accessing antiretroviral therapy (ART), up from 7.7 million in 2010 [1]. However, the development and transmission of antiretroviral (ARV) drug resistance pose significant challenges to HIV care [2]. Pretreatment drug resistance (PDR) refers to resistance that is detected among ARV-naïve people or people with prior ARV drug exposure [3]. The occurrence of PDR greatly decreases the effectiveness of antiviral therapy and is especially associated with virologic failure (VF) and immunologic failure (IF) [4]. Previous studies indicated that individuals who developed PDR were 2–3 times more likely to experience VF within 12 months of treatment, which resulted in reduced long-term effectiveness of first-line therapy [5]. Additionally, the failure to adjust ART regimens based on DR during treatment may worsen this issue, which emphasizes the importance of tailoring ART according to pretreatment genotypic resistance.

*HIV-1* genotype resistance testing can potentially reduce treatment failure and curb the transmission of new infections. However, ART regimens are primarily determined by national guidelines in China, with limited consideration of potential drug resistance at the initiation of ART [6]. Due to limited resources, routine *HIV-1* genotype resistance testing before ART initiation is not commonly practiced in China [7]. Alarmingly, PDR is becoming increasingly severe. Recently, a nationwide survey reported that the PDR prevalence has rapidly risen from 6.8% in 2017 to 7.4% in 2022 in China [7,8]. The latest studies have shown that PDR has reached a medium level in many regions of China, such as Shanghai (17.4%) [9], Tianjin (11.5%) [10], Liangshan (12.2%) [8], Yunnan (9.3%) [8]. These data suggest that surveillance of HIV-1 drug resistance is essential.

The global increase in drug resistance to non-nucleoside reverse-transcriptase inhibitors (NNRTIs) is concerning, particularly in China, highlighting the urgent need for action to address this issue. According to the 2021 World Health Organization (WHO) *HIV* drug resistance report, more than 70% (21/30) of the countries reporting data from 2014 to 2020 recorded primary NNRTI resistance rates above 10% among adults starting ART [11]. In particular, resistance to NNRTIs in China increased from 2.15% to 3.81% from 2012 to 2017 [12]. These data indicate a significant threat to the effectiveness of NNRTI-based ART. Thus, according to the WHO surveillance guidelines, it is a top priority for countries using efavirenz (EFV) or nevirapine (NVP) for first-line ART to implement PDR surveillance [13]. Additionally, the WHO updated its ART guidelines in 2019 to recommend prioritizing the use of non-NNRTI-containing regimens [14].

Given the increasing resistance to NNRTIs, prioritizing the adoption of alternative regimens with lower resistance potential has become imperative. Integrase strand-transfer inhibitors (INSTIs) are a new class of drugs that target *HIV-1* integrase, offering innovative ART regimens for patients. INSTI-based regimens are recommended by the International Antiviral Society—USA (IAS-USA) for most patients owing to their high effectiveness, tolerability, safety, and high barrier to resistance [15]. However, despite the growing use of INSTIs in first-line ART, drug resistance may still occur. According to a 2024 WHO survey, the overall prevalence of PDR to INSTIs was very low (≤0.4%). Among the data reported from 10 countries, South Sudan reported a 0.2% prevalence of dolutegravir (DTG) resistance due to the rare non-polymorphic integrase mutation *S153F/Y* [16]. In China, the major prevalence of INSTI resistance among treatment-naïve patients was recorded in Guangdong (2.65%) [17], Jiangsu (1.7%) [18], and Henan (1.2%) [19]. According to the latest WHO guidelines, as countries scale up the transition to INSTIs, surveillance for drug resistance among people for whom INSTI-containing regimens are failing in low- and middle-income countries will be required [16]. Given the increasing use of INSTIs in Guangxi and the global demand for monitoring INSTI resistance, initiating PDR surveillance for INSTIs in this region is crucial.

Guangxi, in southwestern China, is one of the regions with the most severe HIV/AIDS epidemic in China [20,21]. As of October 2023, about 120,000 individuals with HIV/AIDS were living in the province, with around 1000 new cases reported annually. Among these, Nanning City had the largest number of AIDS cases in Guangxi [22]. However, limited data are available on PDR, particularly, data on INSTIs. In this study, we investigated *HIV-1* PDR (including to NRTIs, NNRTIs, PIs, and INSTIs) among ART-naïve patients in Nanning and conducted a one-year follow-up to observe the effectiveness of ART. Additionally, a molecular transmission network analysis was used to identify the PDR clusters and DRM-related transmission. Our study provides comprehensive data on drug resistance and reveals treatment effectiveness at different levels of resistance, further improving the monitoring of PDR.

## 2. Materials and Methods

### 2.1. Study Population

From 2019 to 2023, a total of 249 newly diagnosed HIV-1-infected individuals from Nanning City, Guangxi, were initially screened. Written informed consent was obtained from all participants. Blood samples were collected and then processed in the laboratory. Enrollment required the successful acquisition of *pol* gene sequences and complete patient data. Consequently, 234 participants were successfully enrolled in the analysis. The eligibility criteria were as follows: (1) newly diagnosed HIV-1-infected individuals between May 2019 and December 2023; (2) individuals aged ≥ 18 years; (3) individuals who signed the informed consent for PDR testing; (4) successful collection of blood samples. The exclusion criteria were as follows: (1) individuals who previously received antiretroviral therapy; (2) failure to obtain *pol* sequences, a mixed-based ratio of ≥5%, or presence of stop codons; (3) *HIV-1 pol* sequence that was too short; (4) patients lacking complete information, including socio-demographic or clinical records. Socio-demographic and clinical characteristics were collected, including sex, age, marital status, transmission route, ART regimen, CD4^+^ T cell counts, CD8^+^ T cell counts, and *HIV-1* RNA viral load. Follow-up information was gathered over a nearly one-year period.

### 2.2. Laboratory Testing and Subtyping

*HIV-1* RNA was extracted from plasma with the High Pure Viral RNA Kit (Roche, Mannheim, Germany). Partial *pol* sequences (HXB2 position: 2253–5096) were amplified with the PrimeScript^TM^ One-Step RT-PCR Kit Ver. 2 (Takara, Kodaira, Japan) following the procedures described in a previous study [23]. The positive amplification replicons were purified and sequenced. The chromatogram data were cleaned and assembled using Sequencher 5.4.6 (Gene Codes Corporation, Ann Arbor, MI, USA). The online tool quality control in the Los Alamos National Laboratory HIV database (https://www.hiv.lanl.gov/content/sequence/HIV/mainpage.html, accessed on 10 June 2024) was used to rule out possible cross-contamination. All the nucleotide sequences were also aligned using the online tool HIVAlign (https://www.hiv.lanl.gov/content/sequence/VIRALIGN/viralign.html, accessed on 10 June 2024) and were manually edited using BioEdit 7.0 (Ibis Biosciences, Carlsbad, CA, USA). Then, the online typing tools COMET *HIV-1* (https://comet.lih.lu/, accessed on 11 June 2024) and HIV BLAST (https://www.hiv.lanl.gov/content/sequence/HIV/mainpage.html, accessed on 11 June 2024) were used to determine the *HIV-1* subtype.

### 2.3. Genotypic Resistance Analysis

Drug resistance mutation screening and PDR assessment were conducted using the Stanford University HIV Drug Resistance Database program, version 8.94 (https://hivdb.stanford.edu/, accessed on 11 June 2024). DRMs were classified based on their ability to confer resistance to NRTIs, NNRTIs, PIs, and INSTIs. The participants were stratified into three categories based on their PDR status. No PDR corresponded to Stanford level 1, indicating susceptibility to all prescribed antiretrovirals. Partly active ART referred to Stanford level 2, indicating potential low-level resistance. PDR classified as Stanford levels 3, 4, or 5, reflected low, intermediate, or high resistance to the prescribed antiretrovirals.

### 2.4. Genetic Network Inference

To obtain a high-resolution molecular network, the optimized genetic distance (GD) threshold was selected to identify the largest number of molecular clusters, avoid forming giant clusters, and detect more potential transmission relationships. The optimal GD threshold was defined as the distance that identified the maximum number of transmission clusters (TCs). All *pol* gene sequences were analyzed to determine the optimal GD, which was identified as 0.021. The *HIV-1* genetic network was visualized and analyzed using Cytoscape 3.8.0. A shared DRM was defined as a DRM present in two genetically linked individuals. A PDR-related cluster was defined as a cluster that contained three or more identical DRMs. Large TCs were defined as clusters containing 10 or more individuals.

### 2.5. Statistical Analysis

IF was defined as having a persistent CD4^+^ T cell count below 100 cells/mm^3^ or a recent CD4^+^ T cell count lower than that at baseline. VF was defined as having a viral load higher than 50 copies/mL that was confirmed at the last recording of the viral load (VL) after 24 weeks of initial ART. All information was examined to identify and impute missing data using multiple imputation techniques. Statistical analysis was conducted using the R 4.3.3 and SPSS27.0 software. All collected information was appropriately transformed into categorical variables and described by numbers and percentages. Chi-square and Fisher’s exact tests were used to compare differences between groups. Logistic regression was used to identify the associated factors of PDR. We employed the Cox proportional hazards model to investigate the impact of varying levels of drug resistance on VF and IF, aiming to assess and quantify the effect of resistance on the risk of VF and IF occurrence. Subsequently, all the independent variables of the univariable COX regression analysis were incorporated into a multivariable COX regression model. Crude and adjusted OR, hazard ratios (HR), and 95% CI were calculated. The *p* values were two-sided with a significance level of 0.05. Excel and Graph Pad Prism 9.5 were employed as the primary tools for the figures.

## 3. Results

### 3.1. Socio-Demographic Characteristics and Clinical Characteristics of the Participants

**Figure 1:**
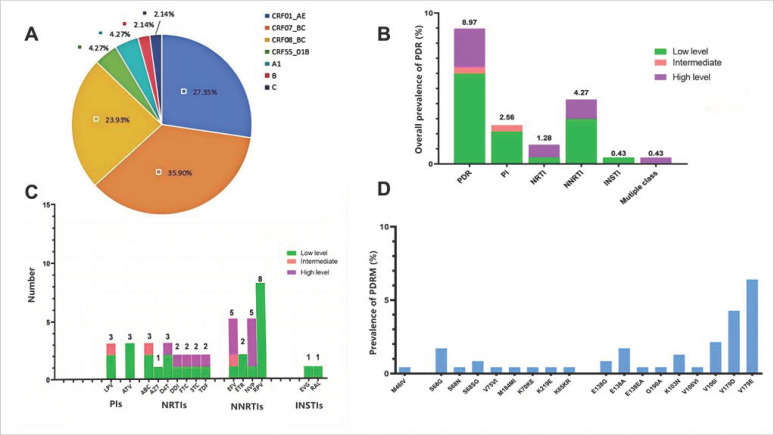
Prevalence and frequency of *HIV-1* drug resistance mutations (DRM) and pretreatment drug resistance (PDR) in Guangxi, Nanning City, 2019–2023. (**A**) Distribution of *HIV-1* genetic subtypes. (**B**) *HIV-1* PDR prevalence by drug class and drug resistance (DR) level. (**C**) Frequency of PDR to 20 *HIV-1* antiretroviral (ARV) drugs. (**D**) *HIV-1* DRM prevalence by drug class. Notes: The estimated level of *HIV-1* resistance to a drug was determined by the Stanford HIVdb program from Sanger sequences. Once the total score was calculated, the estimated level of resistance was calculated as follows: low-level resistance (total score 15 to 29), intermediate resistance (total score 30 to 59), and high-level resistance (total score ≥ 60). Abbreviations: LPV = lopinavir; ATV = atazanavir; ABC = abacavir; AZT = zidovudine; D4T = stavudine; DDI = didanosine; FTC = emtricitabine; 3TC = lamivudine; TDF = tenofovir; EFV = efavirenz; ETR = etravirine; NVP = nevirapine; RPV = rilpivirine; EVG = elvitegravir; RAL = raltegravir.

A total of 249 newly diagnosed HIV-1-infected individuals had PDR testing prior to ART from 2019 to 2023. Seven cases were excluded due to poor sequence quality and the presence of stop codons, five for incomplete gene fragments, and three for missing information. Ultimately, 234 gene sequences and their associated data were included for analysis. The socio-demographic characteristics and clinical characteristics are shown in Table 1. Among the patients, 8.97% (21/234) of them had fully ineffective ART, 12.39% (29/234) showed reduced susceptibility to at least one prescribed drug, and 78.63% (184/234) exhibited no resistance to prescribed drugs. The median (IQR) age was 43 (32–59.75) years. The majority of the participants were male (77.78%), unmarried (46.15%), and lived in urban areas (53.42%). The transmission route was mainly heterosexual contact (70.94%, 166/234), followed by homosexual contact 29.06% (68/234). At ART initiation, 78.21% (183/234) of the participants had a viral load of more than 1000 copies/mL. In addition, CRF07_BC (35.9%, 84/234) and CRF01_AE (27.35%, 64/234) were the main *HIV-1* subtypes, followed by CRF08_BC (23.9%, 56/234), CRF5501_B (4.27%, 10/234), A1(4.27%, 10/234), B (2.14%, 5/234), and C (2.14%, 5/234) (Figure 1A).

Among the three groups categorized by their susceptibility to drug resistance, the majority of the patients were aged over 60 years. The highest predominance of a subtype was identified in the No PDR group (CRF_07BC, 38.59%), followed by the partly active ART group (others, 37.93%) and fully ineffective ART group (CRF08_BC, 33.33%). In terms of marital status, the No PDR group included mostly unmarried individuals (48.91%), while the last two groups included more married or cohabiting individuals, corresponding to 51.72% and 47.62%, respectively. Additionally, the baseline CD4^+^ T cell count was predominantly less than 100 cells/µL in the PDR group, differently from the other two groups. Moreover, no statistically significant differences were observed among the groups in other characteristics, including sex, age, region, baseline CD4/CD8 ratio, baseline VL, and other relevant variables.

### 3.2. Prevalence and Patterns of PDR Across ARV Drug Classes

The overall prevalence of PDR among the patients was 8.97% (21/234). Specifically, the PDR prevalence was 4.27% (10/234) for NNRTIs, 2.56% (6/234) for PIs, 1.28% (3/234) for NRTIs, and 0.43% (1/234) for INSTIs. Most of the PDR was associated with NNRTIs. Furthermore, one patient (0.43%) carried two classes of resistance mutations—specifically, patient 171HSS, with DR to NNRTI (EFV, etravirine (ETR), NVP) and NRTI (zidovudine (AZT), stavudine (D4T)) (Figure 1B). No triple-class resistance was found. The prevalence of PDR in relation to single drugs was the highest for RPV (3.42%, 8/234), NVP (2.14%, 5/234), and EFV (2.14%, 5/234), and the lowest for AZT, elvitegravir (EVG), and raltegravir (RAL), being 0.43% (1/234) for all (Figure 1C).

For NNRTIs, 10 patients showed drug resistance. Eight patients (28.57%, 8/48) showed the highest resistance frequency for low-level resistance to RPV and high-level resistance to NVP (33.33%, 4/12) and EFV (25%, 3/12). Six patients showed resistance to PIs, with five patients who were low-level resistant to tipranavir boosted with atazanavir (ATV) (10.71%, 3/28) and lopinavir (LPV/r)(7.14%, 2/28). The remaining patient had intermediate resistance to LPV/r. Three patients developed resistance to multiple NRTI-related drugs such as abacavir (ABC), D4T, didanosine (DDI), and tenofovir (TDF) combined, which was mostly low-level resistance. One patient (3.57%, 1/28) exhibited low-level resistance to drugs related to INSTIs (EVG and RAL). The resistance levels and frequencies for three different classes of ARV drugs are shown in Figure 1C.

### 3.3. Distribution and Frequency of DRMs

The DRM distribution is depicted in Figure 1D. The prevalence of any DRMs among the 234 patients was 21.37% (50/234). In total, nine NNRTI-associated PDR mutations, eight NRTI-associated PDR mutations, and one PI-associated PDR mutation were identified. Among the NRTI-associated DRMs, *S68G* (1.71%), *S68SG* (0.85%) *S68N* (0.43%), *V75VI* (0.43%), *M184MI* (0.43%), *K70KE* (0.43%), *K219E* (0.43%), and *K65KR* (0.43%) were identified. *S68G* (0.7%) was the most frequent mutation associated with NRTIs. Among the NNRTI-associated DRMs, *V179E* (6.41%), *V179D* (4.27%), *E138A* (1.71%), *E138G* (0.85%), *E138EA* (0.43%), *G190A* (0.43%), *K103N* (1.28%), *V106VI* (0.43%), *V106I* (1.71%), and *V106I* (0.43%) were found. *V179E* (6.41%) and *V179D* (4.27%) were the most predominant mutations among the NNRTI-related DRMs. For the PI-associated DRMs, only the *M46IV* mutation (0.43%) was detected, and no mutation was identified associated with INSTIs.

### 3.4. PDR Transmission Within the HIV-1 Genetic Network

**Figure 2:**
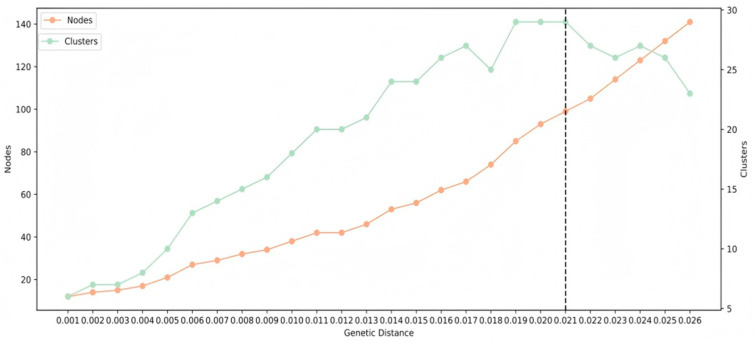
The trend of links and clusters in the genetic network under different genetic distance thresholds. Legend: The genetic distance threshold was based on the Tamura and Nei 93(TN93) model [24]; all of the sample sequences were from patients based in Nanning, Guangxi, diagnosed between 2019 and 2023.

**Figure 3:**
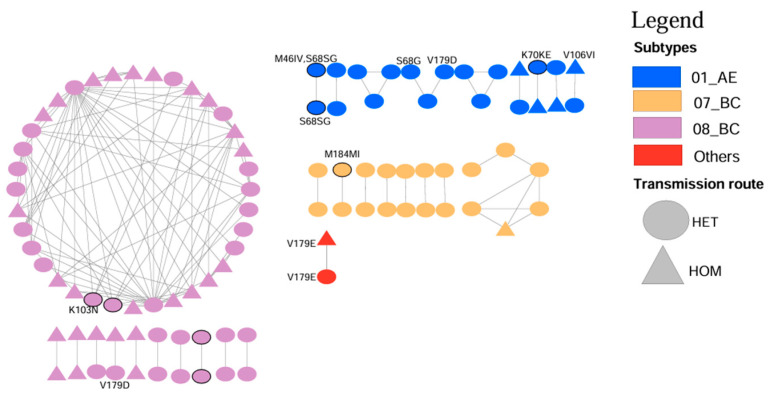
Molecular transmission network among *HIV-1*-positive patients in Nanning, Guangxi, 2019–2023. Notes: Triangles and circles represent different transmission routes, with bold borders indicating drug resistance. Each node is labelled to show clusters of patients with shared DRMs. Different colors represent different *HIV-1* subtypes.

Of the 249 sequences in the PR/RT regions, 15 sequences were removed because they were shorter than 1000 bp, or their number of mixed bases was >5%. Hence, a total of 234 sequences in the PR/RT regions were used to construct a molecular transmission network. Under the optimal genetic distance threshold of 0.01 substitutions per site, the optimal GD was determined to be 0.021, linking 99 sequences (42.3% of the nodes) into 29 clusters (Figure 2). In the molecular transmission network, the highest proportion was observed for CRF08_BC, accounting for 56.5% (56/99). We identified 11 sequences harboring DRM strains across seven distinct clusters, as well as 8 sequences with PDR distributed among five clusters. Most PDR strains linked to clusters were found in male patients (six of eight sequences). The DRM sites identified included *V179E*, *V179D*, and *S68SG*, each at 18.18% prevalence (2/11), and *K103N*, *S68G*, *M46IV*, *V106VI*, *M184MI*, and *K70KE*, each at 9.09% prevalence (1/11) (Figure 3).

### 3.5. Factors Associated with PDR

Univariate and multivariate logistic regression analyses were performed with PDR as the dependent variable. Homosexual patients were significantly less likely to have PDR compared to heterosexual patients (odds ratio [OR] 0.09, 95% CI 0.01–0.86). No statistically significant differences were observed for other variables, including sex, age, marital status, *HIV-1* subtype, baseline CD4^+^ T cell count, viral load, etc. (all *p* > 0.05). These findings suggest that the relationships between these factors and PDR remains inconclusive, highlighting the need for further research or larger sample sizes to validate this association. (Table A1).

### 3.6. Impact of PDR on the Response to ART After One-Year Follow-Up

In a Cox regression multivariate analysis of 234 participants, we assessed the impact of PDR on VF and IF over one year of ART. VF occurred in 19.2% (45/234), and IF in 20.1% (47/234) of the participants. With the No PDR group (n = 174) designated as the reference, the partly active ART group (n = 29) had an adjusted hazard ratio (aHR) of 1.33 (95% CI 0.45–3.97; *p* = 0.608) for VF and 1.44 (0.49–4.18; *p* = 0.506) for IF. The fully ineffective ART group (n = 20) had an aHR of 0.63 (0.16–2.43; *p* = 0.502) for VF and 0.81 (0.28–2.35; *p* = 0.693) for IF. These findings indicated that PDR did not significantly influence the response to ART over one year of follow-up (Table 2).

## 4. Discussion

This study observed a moderate prevalence of PDR, with most cases primarily associated with NNRTIs. This is the first study to evaluate the impact of PDR on the occurrence of virological failure (VF) or immune failure (IF) in patients after one year of treatment initiation. Although drug resistance did not significantly increase the risk of IF and VF after one year of treatment initiation, its potential clinical implications for long-term treatment outcomes warrant attention [25,26]. These findings highlight the critical role of PDR in the management of *HIV* care.

According to the WHO definition, *HIV* drug resistance (DR) prevalence is classified as low (<5%), moderate (5–15%), and high (>15%) [27]. This study found that the prevalence of PDR in Nanning City was 8.97%, slightly higher than that in a previous report for Guangxi (8.3%) [24] and above the national level (7.4%) [28]. Compared to the southwestern provinces of China, such as Chongqing (10.5%) [29], Guangdong (7.4%) [28], and Yunnan (11.4%) [7], the prevalence of PDR in this region remains within the expected range. This prevalence of PDR suggests an increasing trend in PDR over the years, which could be attributed to the spread of drug-resistant strains, inconsistent treatment adherence, or suboptimal management of ART [30].

Notably, the main PDR in Nanning City was to NNRTIs (4.27%), followed by resistance to PIs (2.56%), NRTIs (1.28%), and INSTIs (0.43%). These findings are inconsistent with the national surveillance report, which indicated resistance rates of 1.4% for NRTIs, 1.5% for NNRTIs, and 1.9% for PIs [31]. The highest prevalence of NNRTI resistance in Nanning aligns with the global trend, indicating that NNRTI-resistant strains are the key contributors to the increasing prevalence of PDR [27]. In China, EFV or NVP has been used in first-line ART regimens since 2004. The strong selection pressure from the long-term use of a limited number of NNRTI drugs has facilitated the development and spread of NNRTI-associated drug resistance [32]. Furthermore, the relatively high prevalence of PDR to PIs (2.56%) in this study indicates the widespread use of PI drugs in the region. The increasingly widespread use of PI drugs may have contributed to the increase in PI-associated PDR. This finding suggests that the use of NNRTIs/PIs has significantly contributed to PDR in Guangxi, underscoring the need for continued monitoring of pretreatment resistance.

Unlike ADR, which is primarily driven by DRMs associated with NNRTIs and NRTIs, PDR in Nanning appeared to be mainly driven by NNRTI-associated DRMs. Specifically, the most common NNRTI-associated mutations in this study were *V179D* and *V179E*, which were accompanied by the highest prevalence of PDR to RPV (3.42%, 8/234), EFV (2.13%, 5/234), and NVP (2.13%, 5/234). These findings are consistent with other reports. *V179D/E* caused low-level resistance to RPV but high-level resistance to EFV and NVP, which is similar to the findings from Wenzhou City, China [33]. *K103N* caused high-level resistance to EFV and NVP, consistent with a study from Sapienza University Hospital [34]. The widespread presence of *V179D/E* and *K103N* revealed the regional resistance pattern and has potential implications for treatment strategies, especially regarding the use of EFV and NVP. This may be related to the widespread use of first-line treatment regimens containing EFV or NVP [35] in Guangxi. We also found that all patients carrying E138A/EA/G exhibited low-level resistance to RPV. Additionally, mutations associated with NRTIs, such as *S68G/SG/N*, were frequently observed, though these cases did not show PDR. This finding is inconsistent with other studies [36]. The clinical impact of these mutations on current first-line ART regimens still requires rigorous evaluation.

The prevalence of PDR to PIs in the region has reached 2.56%. PIs have been included in the free ART program in China since 2008 [37,38]. However, the widespread use of PIs could further increase the prevalence of PDR to PIs [29]. In our study, INSTIs exhibit high antiviral potency and high resistance barriers [39]. We observed only two patients exhibiting low-level resistance to EVG and RAL, without any resistant mutations. This suggests that we should consider accelerating the adoption of INSTIs as the first treatment option. Furthermore, the WHO has updated the first-line ART regime to NRTIs plus DTG, especially for PLWH with PDR to EFV or NVP [16].

PDR may be affected by many complex factors. Our study found that patients with heterosexual transmission were more likely to develop PDR than those infected through homosexual transmission, which aligns with a recent survey from Guangxi [40]. Although evidence from China shows an increasing trend for PDR among men who have sex with men (MSM) from 2.5% to 26.9% [41], the prevalence of PDR among MSM in Guangxi remains lower than that among heterosexuals (4.7% vs. 9.3%) [40]. This discrepancy may partly be explained by differences in *HIV-1* subtypes. Although the CRF55_01B and B subtypes are more prone to drug resistance among MSM [42], they represent only 12.9% and 2.2% of the infections, respectively [40], in Guangxi. With the increasing use of PrEP, its impact on PDR has gained growing attention. In recent years, PrEP clinics have been established in Nanning to serve high-risk populations, with approximately 4.9% of Chinese MSM actively receiving PrEP [43,44]. Evidence indicates that appropriate screening and timely initiation of PrEP can reduce the risk of resistance mutations among HIV-1-infected individuals [45]. Particularly, PrEP has demonstrated robust effectiveness among MSM, whereas its effectiveness remains less established in heterosexuals [46]. Additionally, our Cox regression analysis did not show a significant impact of varying resistance levels on long-term treatment outcomes. However, surveillance reports have shown that HIV-1-infected individuals with PDR exhibit a high proportion of VF after ART initiation [47,48], indicating that further long-term studies may provide scientific evidence to guide intervention strategies.

Different *HIV-1* genotypes exhibit varying drug resistances and distinct DRM profiles [12,49]. We observed that CRF08_BC was the predominant *HIV-1* subtype, forming the largest transmission cluster in the genetic network, followed by unique recombinant forms, CRF01_AE, and CRF07_BC. This pattern indicates an increasing trend for the prevalence of CRF08_BC, consistent with a recent survey conducted in Yunnan [50]. The CRF08_BC subtype primarily circulates in the southwestern region and was transmitted into Guangxi in the mid-1990s [51]. In our CRF_08BC cluster, we observed that the transmission pathways between heterosexuals and homosexuals were intertwined, with both groups contributing equally to transmission. This pattern may stem from the high subtype diversity in the region and the long-term accumulation of infections [52]. Specifically, a majority of Chinese MSM conceal their sexual orientation, with many being married [53,54].

The *V179E/D* mutations were the most prevalent within the network, likely due to their high frequency among all DRMs, which aligns with previous findings [24]. The CRF01_AE subtype exhibited the highest number of mutation sites, primarily characterized by S68SG [42]. In contrast, the CRF08-BC cluster only showed the *V179D* and *K103N* mutations, while the CRF07_BC cluster exhibited the M184MI mutation. These mutations—*S68SG*, *K103N*, and *M184MI*—were shared DRMs that may transmit drug resistance [55]. These findings highlight the significant differences in DRMs across subtypes, reflecting the complexity of the resistance mechanisms noted in other studies [2,56]. Overall, routine monitoring of *HIV-1* genotypes is essential for effective prevention and control of the *HIV-1* epidemic.

## 5. Conclusions

In conclusion, our study demonstrates that the prevalence of PDR in Nanning remains below the 10% threshold, suggesting that the current ARV drugs remain effective. NNRTI-associated mutations, particularly *V179D* and *K103N*, predominantly drive resistance patterns and infection transmission. Consequently, there is a compelling need to optimize the ART strategies by considering the incorporation of INSTIs as an alternative to regimens containing EFV, NVP, or RPV. The observed cross-transmission among diverse populations highlights the necessity for targeted interventions, such as increasing the drug resistance testing rate, to mitigate the incidence and further spread of PDR.

## 6. Limitation

This study has several limitations. First, the small sample size may have reduced the statistical power, and the single-center design restricts the generalizability of the findings. Second, the cross-sectional design and relatively short 12-month follow-up difficultly establish causality. Finally, information on prior ARV use, such as pre-exposure prophylaxis or treatment interruptions—factors that could significantly impact resistance—was not collected, reducing the comprehensiveness and interpretive power of the results. These limitations need to be addressed in subsequent studies.

## Figures and Tables

**Table 1:** Socio-demographic characteristics and clinical characteristics of the participants, stratified by their drug resistance level (N, %).

Variables	Total	No PDR	Partly Active ART	Fully Ineffective ART	p *
Total	234 (100.00)	184 (78.63)	29 (12.39)	21 (8.97)	
Sex					
Man	182 (77.78)	150 (81.52)	19 (65.52)	13 (61.9)	0.029
Woman	52 (22.22)	34 (18.48)	10 (34.48)	8 (38.1)	
Age					
<30	48 (20.51)	40 (21.74)	4 (13.79)	4 (19.05)	0.161
30–39	53 (22.65)	41 (22.28)	10 (34.48)	2 (9.52)	
40–49	35 (14.96)	27 (14.67)	2 (6.9)	6 (28.57)	
50–59	37 (15.81)	32 (17.39)	2 (6.9)	3 (14.29)	
≥60	61 (26.07)	44 (23.91)	11 (37.93)	6 (28.57)	
Region					
Urban	125 (53.42)	99 (53.8)	16 (55.17)	10 (47.62)	0.848
Suburban	109 (46.58)	85 (46.2)	13 (44.83)	11 (52.38)	
Marital status					
Unmarried	108 (46.15)	90 (48.91)	10 (34.48)	8 (38.1)	0.588
Married/cohabiting	96 (41.03)	71 (38.59)	15 (51.72)	10 (47.62)	
Divorced/widowed	30 (12.82)	23 (12.5)	4 (13.79)	3 (14.29)	
*HIV* Subtype					
CRF01_AE	64 (27.35)	53 (28.8)	5 (17.24)	6 (28.57)	0.002
CRF07_BC	84 (35.9)	71 (38.59)	8 (27.59)	5 (23.81)	
CRF08_BC	56 (23.93)	44 (23.91)	5 (17.24)	7 (33.33)	
Others	30 (12.82)	16 (8.7)	11 (37.93)	3 (14.29)	
WHO clinical stage at initiation					
1 or 2	145 (61.97)	113 (61.41)	19 (65.52)	13 (61.9)	0.914
3 or 4	89 (38.03)	71 (38.59)	10 (34.48)	8 (38.1)	
TMP SMX use at baseline					
No	189 (80.77)	149 (80.98)	25 (86.21)	15 (71.43)	0.42
Yes	45 (19.23)	35 (19.02)	4 (13.79)	6 (28.57)	
Transmission route					
HET	166 (70.94)	123 (66.85)	23 (79.31)	20 (95.24)	0.014
HOM	68 (29.06)	61 (33.15)	6 (20.69)	1 (4.76)	
Initial ART regimen					
EFV/ANV-based	128 (54.70)	104 (56.52)	19 (65.52)	5 (23.81)	0.015
BIC-based	48 (20.51)	40 (21.74)	2 (6.90)	6 (28.57)	
DTG-based	40 (17.09)	30 (16.3)	4 (13.79)	6 (28.57)	
LPV/r-based	18 (7.69)	10 (5.43)	4 (13.79)	4 (19.05)	
ART regimen change					
No	174 (74.36)	138 (75)	18 (62.07)	18 (85.71)	0.153
Yes	60 (25.64)	46 (25.00)	11 (37.93)	3 (14.29)	
Baseline CD4^+^ T cell count, cell/µL					
<100	61 (26.07)	46 (25.00)	6 (20.69)	9 (42.86)	0.187
100–200	43 (18.38)	33 (17.93)	8 (27.59)	2 (9.52)	
200–349	66 (28.21)	51 (27.72)	11 (37.93)	4 (19.05)	
≥349	64 (27.35)	54 (29.35)	4 (13.79)	6 (28.57)	
Baseline CD8^+^ T cell count					
<1000 cells/μL	139 (59.4)	105 (57.07)	19 (65.52)	15 (71.43)	0.345
≥1000 cells/μL	95 (40.6)	79 (42.93)	10 (34.48)	6 (28.57)	
Baseline CD4/CD8 ratio					
<0.5	195 (83.33)	153 (83.15)	26 (89.66)	16 (76.19)	0.447
≥0.5	39 (16.67)	31 (16.85)	3 (10.34)	5 (23.81)	
Baseline VL					
<1000 copies/mL	51 (21.79)	35 (19.02)	8 (27.59)	8 (38.10)	0.097
≥1000 copies/mL	183 (78.21)	149 (80.98)	21 (72.41)	13 (61.90)	

Note. * Chi-square trend test or Fisher’s exact test.

**Table 2:** Impact of pretreatment drug resistance on virological failure and immunological failure after one-year follow-up.

Variables	Total	VF			IF		
		n (%)	aHR (95CI)	*p*-Value	n (%)	aHR (95CI)	*p*-Value
Group	234 (100.00)	45 (19.23)			47 (20.09)		
No PDR (reference)	174 (78.03)	37 (21.26)	1	-	37 (21.26)	1	-
Partly active ART	29 (13.00)	5 (17.24)	1.33 (0.45, 3.97)	0.608	5 (17.24)	1.44 (0.49, 4.18)	0.506
Fully ineffective ART	20 (8.97)	3 (15.00)	0.63 (0.16, 2.43)	0.502	5 (25)	0.81 (0.28, 2.35)	0.693

Note. Adjusted variables: sex, age, region, marital status, *HIV-1* subtype, WHO clinical stage, TMP SMX use at baseline, transmission route, initial ART regimen, ART regimen change, baseline CD4^+^ T cell count, baseline CD8^+^ T cell count, baseline CD4/CD8 ratio, baseline VL.

## Data Availability

The datasets are available from the corresponding author on reasonable request.

## Abbreviations

The following abbreviations are used in this manuscript:

HIV	Human immunodeficiency virus type 1
PDR	Pretreatment drug resistance
DR	Drug resistance
ADR	Acquired drug resistance
DRM	Drug resistance mutation
WHO	World Health Organization
ART	Antiretroviral therapy
ARV	Antiretroviral
MSM	Men who have sex with men
VF	Virological failure
IF	Immunological failure
TCs	Transmission clusters
VL	Viral load
GD	Genetic distance
NNRTIs	Non-nucleoside reverse-transcriptase inhibitors
NRTIs	Nucleoside reverse-transcriptase inhibitors
PIs	Protease inhibitors
INSTIs	Integrase strand-transfer inhibitors
DRV	Darunavir
FPV	Fosamprenavir
IDV	Indinavir
LPV	Lopinavir
NFV	Nelfinavir
SQV	Saquinavir
TPV	Tipranavir
ABC	Abacavir
AZT	Zidovudine
D4T	Stavudine
DDI	Didanosine
FTC	Emtricitabine
3TC	Lamivudine
TDF	Tenofovir
DOR	Doravirine
EFV	Efavirenz
ETR	Etravirine
NVP	Nevirapine
RPV	Rilpivirine
BIC	Bictegravir
CAB	Cabotegravir
DTG	Dolutegravir
EVG	Elvitegravir
RAL	Raltegravir
TMP/SMX	Trimethoprim/sulfamet
OR	Odds ratio
aOR	Adjusted odds ratio
HR	Hazard ratio
aHR	Adjusted hazard ratio
CI	Confidence interval
ART	Antiretroviral therapy
ARV	Antiretroviral

## Appendix A

**Table A1:** Logistic regression analysis for factors affecting patients with PDR.

Variables	Total	PDR	OR (95%CI)	*p*	aOR (95%CI)	*p*
Sex						
Female	182 (77.78%)	13 (7.14%)				
Male	52 (22.22%)	8 (15.38%)	2.36 (0.92, 6.06)	0.073	1.7 (0.54, 5.35)	0.362
Age						
<30	48 (20.51%)	4 (8.33%)				
30–39	53 (22.65%)	2 (3.77%)	0.43 (0.08, 2.47)	0.345	0.25 (0.03, 1.91)	0.181
40–49	35 (14.96%)	6 (17.14%)	2.28 (0.59, 8.77)	0.232	1.41 (0.21, 9.51)	0.722
50–59	37 (15.81%)	3 (8.11%)	0.97 (0.20, 4.63)	0.97	0.54 (0.06, 5.34)	0.6
≥60	61 (26.07%)	6 (9.84%)	1.2 (0.32, 4.52)	0.788	0.59 (0.07, 4.93)	0.629
Region						
Urban	109 (46.58%)	11 (10.09%)				
Suburban	125 (53.42%)	10 (8%)	0.77 (0.32, 1.9)	0.577	0.51 (0.17, 1.47)	0.209
Marital status						
Unmarried	108 (46.15%)	8 (7.41%)				
Married or cohabiting	96 (41.03%)	10 (10.42%)	1.45 (0.55, 3.85)	0.451	0.84 (0.16, 4.29)	0.834
Divorced or widowed	30 (12.82%)	3 (10%)	1.39 (0.34, 5.59)	0.644	0.55 (0.07, 4.21)	0.568
Subtype						
CRF01_AE	64 (27.35%)	6 (9.38%)				
CRF07_BC	84 (35.9%)	5 (5.95%)	0.61 (0.18, 2.1)	0.435	0.66 (0.17, 2.61)	0.552
CRF08_BC	56 (23.93%)	7 (12.5%)	1.38 (0.44, 4.38)	0.584	0.93 (0.24, 3.62)	0.915
Others	30 (12.82%)	3 (10%)	1.07 (0.25, 4.62)	0.924	0.7 (0.12, 3.97)	0.684
WHO clinical stage at initiation						
1 or 2	145 (61.97%)	13 (8.97%)				
3 or 4	89 (38.03%)	8 (8.99%)	1 (0.40, 2.52)	0.995	0.56 (0.17, 1.85)	0.339
TMP SMX use at baseline						
No	189 (80.77%)	15 (7.94%)				
Yes	45 (19.23%)	6 (13.33%)	1.78 (0.65, 4.89)	0.26	1.03 (0.18, 5.95)	0.973
Transmission route						
Heterosexual	166 (70.94%)	20 (12.05%)				
Homosexual	68 (29.06%)	1 (1.47%)	0.11 (0.01, 0.83)	0.032	0.09 (0.01, 0.86)	0.037
Baseline CD4^+^ T cell count, cell/µL						
<100	61 (26.07%)	9 (14.75%)				
100–200	43 (18.38%)	2 (4.65%)	0.28 (0.06, 1.38)	0.118	0.25 (0.03, 2.06)	0.199
200–349	66 (28.21%)	4 (6.06%)	0.37 (0.11, 1.28)	0.117	0.29 (0.04, 2.13)	0.223
≥349	64 (27.35%)	6 (9.38%)	0.6 (0.20, 1.79)	0.359	0.38 (0.05, 3.16)	0.371
Baseline CD8^+^ T cell count						
<1000 cells/μL	139 (59.4%)	15 (10.79%)				
≥1000 cells/μL	95 (40.6%)	6 (6.32%)	0.56 (0.21, 1.49)	0.245	0.69 (0.19, 2.56)	0.584
Baseline CD4/CD8 ratio						
<0.5	195 (83.33%)	16 (8.21%)				
≥0.5	39 (16.67%)	5 (12.82%)	1.65 (0.56, 4.79)	0.361	1.8 (0.39, 8.35)	0.45
Baseline VL						
<1000 copies/mL	51 (21.79%)	8 (15.69%)				
≥1000 copies/mL	183 (78.21%)	13 (7.1%)	0.41 (0.16, 1.05)	0.064	0.49 (0.16, 1.45)	0.197

**Table A2:** Cox proportional hazards analysis of immunologic and virologic failure in patients approximately one year after ART initiation.

Variables	Total (N = 223)	VF			IF		
		n = 45	aHR [95%CI]	*p*	n = 47	aHR [95%CI]	*p*
Group							
No PDR	174 (78.03%)	37 (21.26%)			37 (21.26%)		
Partly active ART	29 (13%)	5 (17.24%)	1.33 [0.45, 3.97]	0.608	5 (17.24%)	1.44 [0.49, 4.18]	0.506
Fully active ART	20 (8.97%)	3 (15.00%)	0.63 [0.16, 2.43]	0.502	5 (25%)	0.81 [0.28, 2.35]	0.693
Sex							
Woman	172 (77.13%)	38 (22.09%)			38 (22.09%)		
Man	51 (22.87%)	7 (13.73%)	0.7 [0.28, 1.78]	0.456	9 (17.65%)	0.89 [0.35, 2.24]	0.805
Age							
<30	45 (20.18%)	14 (31.11%)			12 (26.67%)		
30–39	52 (23.32%)	6 (11.54%)	0.12 [0.04, 0.37]	<0.001	11 (21.15%)	0.62 [0.23, 1.65]	0.337
40–49	34 (15.25%)	6 (17.65%)	0.26 [0.07, 0.9]	0.034	10 (29.41%)	0.81 [0.27, 2.42]	0.705
50–59	34 (15.25%)	6 (17.65%)	0.15 [0.04, 0.61]	0.008	8 (23.53%)	1.1 [0.32, 3.79]	0.885
≥60	58 (26.01%)	13 (22.41%)	0.27 [0.07, 1.05]	0.058	6 (10.34%)	0.49 [0.13, 1.9]	0.303
Region							
Urban	105 (47.09%)	22 (20.95%)			25 (23.81%)		
Suburban	118 (52.91%)	23 (19.49%)	0.69 [0.36, 1.32]	0.259	22 (18.64%)	0.71 [0.37, 1.37]	0.311
Marital status							
Unmarried	101 (45.29%)	21 (20.79%)			27 (26.73%)		
Married or cohabiting	93 (41.7%)	18 (19.35%)	2.38 [0.77, 7.36]	0.133	18 (19.35%)	0.68 [0.25, 1.79]	0.43
Divorced or widowed	29 (13%)	6 (20.69%)	1.81 [0.44, 7.47]	0.412	2 (6.9%)	0.19 [0.03, 1.04]	0.055
Subtype							
CRF01_AE	56 (25.11%)	8 (14.29%)			12 (21.43%)		
CRF07_BC	82 (36.77%)	22 (26.83%)	1.3 [0.53, 3.22]	0.57	16 (19.51%)	0.62 [0.26, 1.48]	0.28
CRF08_BC	56 (25.11%)	12 (21.43%)	1.04 [0.38, 2.89]	0.936	14 (25%)	1 [0.41, 2.46]	0.999
Others	29 (13%)	3 (10.34%)	0.25 [0.06, 1.09]	0.064	5 (17.24%)	0.88 [0.26, 2.92]	0.832
WHO clinical stage							
1 or 2	139 (62.33%)	32 (23.02%)			28 (20.14%)		
3 or 4	84 (37.67%)	13 (15.48%)	0.36 [0.16, 0.78]	0.009	19 (22.62%)	1.13 [0.56, 2.3]	0.736
TMP SMX use at baseline							
No	180 (80.72%)	36 (20%)			39 (21.67%)		
Yes	43 (19.28%)	9 (20.93%)	0.29 [0.09, 0.98]	0.046	8 (18.6%)	0.4 [0.12, 1.38]	0.146
Transmission route							
Heterosexual	158 (70.85%)	29 (18.35%)			32 (20.25%)		
Homosexual	65 (29.15%)	16 (24.62%)	1.49 [0.65, 3.4]	0.348	15 (23.08%)	0.76 [0.33, 1.75]	0.521
Initial ART regimen							
EFV/ANV-based	122 (54.71%)	25 (20.49%)			24 (19.67%)		
BIC-based	45 (20.18%)	9 (20%)	0.93 [0.39, 2.23]	0.873	15 (33.33%)	2.37 [1.07, 5.25]	0.034
DTG-based	38 (17.04%)	8 (21.05%)	1.06 [0.41, 2.71]	0.909	7 (18.42%)	1.34 [0.52, 3.41]	0.544
Lpv/r-based	18 (8.07%)	3 (16.67%)	1.1 [0.31, 3.97]	0.881	1 (5.56%)	0.7 [0.08, 5.86]	0.743
ART regimen change							
No	166 (74.44%)	30 (18.07%)			31 (18.67%)		
Yes	57 (25.56%)	15 (26.32%)	1.81 [0.89, 3.68]	0.104	16 (28.07%)	1.59 [0.74, 3.39]	0.231
Baseline CD4, cell/µL							
<100	59 (26.46%)	18 (30.51%)			14 (23.73%)		
100–200	42 (18.83%)	6 (14.29%)	0.11 [0.03, 0.36]	<0.001	2 (4.76%)	0.09 [0.02, 0.5]	0.006
200–349	61 (27.35%)	7 (11.48%)	0.06 [0.02, 0.2]	<0.001	10 (16.39%)	0.19 [0.05, 0.66]	0.009
≥349	61 (27.35%)	14 (22.95%)	0.24 [0.07, 0.82]	0.023	21 (34.43%)	0.3 [0.08, 1.08]	0.065
CD4/CD8 at baseline							
<0.5	134 (60.09%)	27 (20.15%)			25 (18.66%)		
≥0.5	89 (39.91%)	18 (20.22%)	0.72 [0.32, 1.62]	0.425	22 (24.72%)	1.84 [0.8, 4.25]	0.15
Baseline CD8 count							
<1000 cells/μL	185 (82.96%)	39 (21.08%)			32 (17.3%)		
≥1000 cells/μL	38 (17.04%)	6 (15.79%)	0.61 [0.21, 1.77]	0.364	15 (39.47%)	2.23 [0.91, 5.49]	0.081
Baseline *HIV-1* RNA							
<1000 copies/mL	48 (21.52%)	9 (18.75%)			16 (33.33%)		
≥1000 copies/mL	175 (78.48%)	36 (20.57%)	2.71 [1.14, 6.44]	0.024	31 (17.71%)	0.58 [0.29, 1.18]	0.134
